# Draft Genome Sequence of *Staphylococcus carnosus* subsp. *utilis* LTH 7013, Isolated from South Tyrolean Ham

**DOI:** 10.1128/genomeA.00456-15

**Published:** 2015-05-14

**Authors:** Anne Müller, Christopher Huptas, Mareike Wenning, Herbert Schmidt, Agnes Weiss

**Affiliations:** a Department of Food Microbiology and Hygiene, Institute of Food Science and Biotechnology, University of Hohenheim, Stuttgart, Germany; b Lehrstuhl für Mikrobielle Ökologie, Zentral-institut für Ernährungs- und Lebensmittel-forschung (ZIEL), Wissenschaftszentrum Weihenstephan, Technische Universität München, Freising, Germany

## Abstract

*Staphylococcus carnosus* is used as a starter culture in meat fermentation, where it contributes to color formation and produces aromatic compounds. Here, we report the first draft genome sequence of an *S. carnosus* subsp. *utilis* strain, LTH 7013, isolated from South Tyrolean ham, with potential application as a starter culture.

## GENOME ANNOUNCEMENT

Strains of the species *Staphylococcus carnosus* are used as bacterial starter cultures for the fermentation of meat products, such as sausage. They contribute to the development and stabilization of a typically red color (1) and to the formation of aromatic compounds (2). This species consists of two subspecies, namely, *S. carnosus* subsp. *carnosus* and *S. carnosus* subsp. *utilis*. The complete genome sequence of *S. carnosus* subsp. *carnosus* strain TM300 has been published (3), but no insights into the genome sequence of *S. carnosus* subsp. *utilis* have been reported so far.

Strain LTH 7013 was isolated from South Tyrolean ham, and genomic DNA was extracted with CTAB (4). Sequencing was performed in the 250-nucleotide (nt) paired-end mode on an Illumina MiSeq platform using the TruSeq DNA PCR-free sample prep kit (Illumina) for library preparation. Sequenced read pairs were trimmed by 10 nt from the 5′ end and 1 nt from the 3′ end prior to read filtering (parameter setting, −l 70 −s 20) with the NGS QC toolkit version 2.2.3 (5). Then, high-quality read pairs were assembled with SPAdes version 2.5.1 (6) using default parameter settings.

The draft genome sequence of *S. carnosus* subsp. *utilis* LTH 7013 comprises 34 contigs with a total length of 2,632,443 bp. The contigs were compared to the genome sequence of strain TM300 (3) with GGDC version 2.0 (7), which confirmed the classification to the subspecies *utilis*. The annotation with RAST (8) predicted 2,607 coding sequences (CDSs) and 68 RNAs. The phage search tool PHAST (9) revealed one intact prophage region with a size of 32.1 kb and a GC content of 34%.

The draft genome of LTH 7013 reveals a multitude of functions necessary for a starter culture. For example, the pathway for reduction of nitrate to nitrite and further to ammonia is complete. Nitrate reductase activity contributes to the formation of red color in meat products and is therefore a welcome characteristic for a starter culture. Also, pathways for the degradation of different amino acids like isoleucine and valine are complete. Degradation products of both amino acids are described to have a sensory effect (10). Similar to *S. carnosus* subsp. *carnosus*, *S. carnosus* subsp. *utilis* has different possibilities to respond to osmotic, cold, and oxidative stress, for example, the glycine betaine transporter OpuD, the cold shock protein CspA, or genes encoding for superoxide dismutase and catalase. No genes encoding for staphylococcal toxins and superantigens were predicted. One gene, which encodes for a beta-lactamase, was found in the genome but not on the intact prophage.

### Nucleotide sequence accession numbers. 

This whole-genome shotgun project has been deposited in GenBank under the accession number LAIU00000000. The version described in this paper is the version LAIU00000000.1.
